# Day-to-day variability and method agreement of sweat sodium concentration during pilocarpine iontophoresis and exercise

**DOI:** 10.1007/s00421-026-06409-0

**Published:** 2026-08-27

**Authors:** Shawn C. Wierick, Zachary J. McKenna, Rosie I. Perez, Xiujing Zhao, Ryan S. Sellner, Becca M. Allen, Brendon P. McDermott

**Affiliations:** 1 Heat & Hydration Optimization (H 2 O) Laboratory, Exercise Science Research Center, University of Arkansas, Fayetteville, AR 72701 USA; 2 Department of Health, Human Performance, and Recreation, University of Arkansas, Fayetteville, AR 72701 USA

**Keywords:** Eccrine sweat, Sodium balance, Hydration

## Abstract

**Purpose:**

Sweat sodium testing is increasingly used to estimate electrolyte losses during exercise; however, the reliability of sweat sodium assessment and the influence of methodological factors remain unclear. We evaluated the day-to-day variability and agreement between common sweat induction, collection, and analytical approaches, including pilocarpine iontophoresis and exercise-induced sweating.

**Methods:**

Twenty physically active adults (10 males 33 ± 11 y, 10 females 33 ± 10 y) completed two experimental trials separated by 48 h. Local sweat was induced at rest using pilocarpine iontophoresis. Participants then performed 60 min of treadmill running or cycling in 27 °C, 50% relative humidity, during which sweat was collected. Sweat sodium concentration was then analyzed and agreement between measurements was evaluated using Bland-Altman analysis.

**Results:**

Day-to-day variability was good, with coefficients of variation of 13.8% for iontophoresis, 14.9% for exercise-derived Macroduct samples, and 9.9% for patch-based collection. Iontophoresis-derived sweat sodium concentrations were higher than exercise-derived values (42.0 ± 9.3 vs. 33.5 ± 11.0 mmol·L^− 1^) with a mean bias of -8.5 mmol·L^− 1^ (level of agreement (LoA): -32.2 to 15.2).

**Conclusion:**

Sweat sodium concentration exhibits moderate day-to-day variability under controlled laboratory conditions. Exercise-derived values were lower than those obtained via pilocarpine iontophoresis, with iontophoresis overestimating sweat sodium by ~ 8.5mmol·L^− 1^ (~ 195mg·L^− 1^) on average. Although differences in methodological approaches were relatively small, these methodological factors contribute additional variability. Collectively, these findings indicate that both biological and methodological factors contribute to variability in sweat sodium assessment, and iontophoresis-derived values should be interpreted cautiously when estimating sodium losses during exercise.

**Supplementary Information:**

The online version contains supplementary material available at https://doi.org/10.1007/s00421-026-06409-0.

## Introduction

Prolonged exercise, particularly when performed in hot and/or humid conditions, places significant strain on the thermoregulatory system to maintain a stable core temperature. This increases sweating and skin blood flow to promote heat dissipation(Nadel et al. 1971; Rowell et al. 1970). Indeed, whole body sweat rates commonly exceed 1 L·h^− 1^ in endurance athletes, with reported ranges spanning from 0.5 to > 2.5 L·h^− 1^, reflecting substantial inter-individual variability (Barnes et al. 2019). As a result, endurance athletes are challenged with maintaining fluid and electrolyte balance during prolonged exercise. Sodium, the primary determinant of plasma osmolality and the predominant electrolyte lost in sweat, is perhaps the most critical electrolyte to regulate during endurance exercise (Baker and Wolfe 2020). In endurance athletes, sweat sodium concentrations are typically near 40 mmol·L^− 1^ during exercise (Barnes et al. 2019), though considerable variability has been reported in the literature with whole body values ranging from 10 to 70 mmol·L^− 1^ (Baker 2017; Sawka et al. 2007). When sustained over several hours of continuous exercise, these sweat losses can result in large cumulative sodium losses that, if not addressed, may disrupt fluid balance (Sawka et al. 2007; Hew-Butler et al. 2015), thermoregulation (Periard et al. 2021; Sawka et al. 2007), and contribute to altered neuromuscular function (Schwellnus 2009). Inappropriate replacement of fluid and sodium during extended periods of exercise has been associated with exercise associated hyponatremia, gastrointestinal distress, muscle cramping, nausea, and cognitive impairment (Hew-Butler et al. 2015; Costa et al. 2017; Schwellnus 2009). Likewise, overconsumption of sodium during exercise may result in gastrointestinal distress and/or delayed gastric emptying(Sugihara et al. 2014). Although these consequences can occur across a range of endurance disciplines, ultra-endurance athletes are particularly vulnerable due to the cumulative nature of losses and the logistical challenges of replacement (i.e., extended race duration, aid-station spacing, variable terrain, course layout, and fluctuating environmental conditions) (Wierick et al. 2025; Hoffman and Stuempfle 2014).

To overcome these challenges, endurance athletes and coaches have turned to individualized sweat testing to inform fluid and electrolyte prescriptions (Baker 2017; Wierick et al. 2025). Pharmacological sweat induction via pilocarpine iontophoresis, a technique that was standardized decades ago for clinical sweat chloride diagnostics (i.e., cystic fibrosis), has recently been adopted in athletic settings due to its speed, portability, and lack of exercise or thermal stress requirements (Gibson and Cooke 1959; Gokdemir and Karadag 2021). With iontophoresis, a mild electrical current delivers pilocarpine to stimulate eccrine glands locally, enabling sweat collection at rest. The practical appeal is clear; however, key questions remain regarding translation to exercise and heat stress. While pilocarpine evokes cholinergic activation locally(Gibson and Cooke 1959), prolonged exercise recruits a broader response integrating central and peripheral thermoregulatory drives (Shibasaki et al. 2006; Gibson and Cooke 1959). Thus, it remains unclear whether pilocarpine iontophoresis provides an accurate estimate of exercise-induced sweat sodium loss.

Sweat sodium concentration can vary substantially between individuals due to factors such as sex, heat acclimation, body size, training status, and environmental conditions (Barnes et al. 2019; Baker 2017; Kirby and Convertino 1986). In addition to these between-individual differences, sweat sodium concentration also exhibits normal intra-individual day-to-day variability. For example, whole-body sweat sodium concentration has been reported to vary by approximately 11–17% even under standardized testing conditions and may be further influenced by short-term changes in dietary sodium intake (Baker 2017; McCubbin et al. 2019). Methodological factors, including sweat collection approach, anatomical sampling site, sample handling, and analytical technique, can further contribute to measurement variability (Baker 2017; Barnes et al. 2019). Together, these sources of biological and methodological variability complicate the assumption that a single iontophoresis-derived value can reliably substitute for exercise-derived measurements when prescribing sodium replacement during training or competition.

A recent study directly compared pilocarpine-induced and exercise-induced sweat sodium concentrations across three separate 20-minute bouts of exercise under thermoneutral laboratory conditions (~ 20 °C, ~ 35% relative humidity) at low-, moderate-, and high intensity in trained endurance athletes (Harris et al. 2026). The authors reported an intensity-dependent agreement between pilocarpine and exercise-derived sweat sodium, with strong agreement observed at a moderate-intensity whereas sweat sodium concentration with pilocarpine was higher when compared to low-intensity exercise and lower compared with high-intensity (Harris et al. 2026). Importantly, pilocarpine-derived sweat sodium demonstrated low intra-individual variability (CV: 5.5%) across repeated testing sessions under standardized conditions (Harris et al. 2026). While these findings provide important insight into the physiological relationship between pilocarpine- and exercise-induced sweating, several practical considerations relevant to applied sweat testing remain unresolved. The reliability of repeated sweat sodium measurements and the influence of methodological factors, such as sweat collection technique and analytical device selection, are particularly important because these factors may contribute additional varaibility beyond normal biological fluctuations. Understanding the magnitude of these sources of variability is therefore critucal for athletes, coaches, sport scientists, and practitioners who use sweat testing to guide indiviudalized hydration and sodium replacment strategies. Furthermore, it remains unclear whether differences between pilocarpine- and exercise-derived sweat sodium concnetrations are large enough to meangingully affect individualized hydration and sodium replacement strategies during endurance exercise.

Accordingly, the purpose of this study was to evaluate the reliability of sweat sodium assessment and determine the influence of common sweat induction, sweat collection, and analytical approaches on sweat sodium concentrations in physically active adults. Specifically, we (1) determined the day-to-day variability of sweat sodium conccntration obtained via pilocarpine iontophoresis and exercise-induced sweating, (2) compared sweat sodium concentrations obtained via pilocarpine iontophoresis with those measured during exercise, and (3) evaluated agreement between two sweat collection methods (patch and Macroduct) and two analytical devices (EasyLyte and Wescor). The findings from this study provided practical information regarding the physiological, analytical, and methodological sources of variability in sweat sodium assessment and may help athletes, coaches, sport scientists, and practitioners interpret sweat sodium test results and develop individualized hydration and sodium replacement strategies.

## Methods

### Participants

Twenty physically active adults (10 males: 33 ± 11 y; 10 females: 33 ± 10 y) volunteered to participate in this study. Participant characteristics are presented in Table 1. Recruitment occurred through university announcements, local sports clubs, and word of mouth. All participants reported engaging in structured endurance training more than three times per week and were free from any medical conditions or medications known to influence thermoregulation, fluid balance, or exercise tolerance. Written informed consent was obtained prior to participation, and the protocol was approved by the University Institutional Review Board.

**Table 1:** Baseline characteristics of participants. Values are presented as mean ± SD

	All	Males	Females
Age (years)	33 ± 11	33 ± 10	33 ± 10
Weight (kg)	71.1 ± 14.8	82.1 ± 12.4	60.2 ± 6.8
Height (cm)	171.1 ± 11.0	180.2 ± 6	162.1 ± 5
Body mass index (kg/m^2^)	24.1 ± 2.8	25.2 ± 2.8	22.9 ± 2.3
*Pre urine specific gravity*			
Trial 1	1.011 ± 0.01	1.011 ± 0.01	1.010 ± 0.01
Trial 2	1.011 ± 0.01	1.011 ± 0.01	1.010 ± 0.01
*24 h fluid intake (L)*			
Trial 1	4.2 ± 1.4	4.1 ± 1.4	4.3 ± 1.5
Trial 2	3.9 ± 1.5	4.2 ± 1.7	3.6 ± 1.4
*24 h dietary sodium (mg)*			
Trial 1	3585 ± 1878	3562 ± 1892	3609 ± 1966
Trial 2	3403 ± 1785	3410 ± 2226	3398 ± 1472
*24 h dietary carbohydrate (g)*			
Trial 1	241 ± 100	284 ± 101	197 ± 81
Trial 2	205 ± 56	236 ± 53	180 ± 47

### Experimental design

A repeated measures design was used to assess the agreement and day-to-day variability between iontophoresis-based and exercise-based measures of sweat sodium concentration. Each participant completed two trials separated by 48 h. Trials were scheduled at the same time of day to avoid diurnal variation. Participants were instructed to replicate their habitual diet and fluid intake for 24 h before each session and to consume approximately 500 mL of water on the evening prior and morning of each trial. Diet and fluid logs were recorded using nutrition tracking software (Nutritionist Pro, Axxya Systems LLC, Redmond, WA). To obtain exercise based sweat sodium concentrations participants performed 60-minutes of exercise on either a treadmill (Proform, Boston Marathon 4.0, Logan UT) or cycling ergometer (Velotron Racermate, Seattle, WA). Exercise modality was selected based on training background and preference but replicated within participants across trials for consistency. A 60-minute exercise duration was selected to provide sufficient time to establish sustained sweating and obtain adequate sweat volume for comparison of the different sweat collection and analytical methods under standardized laboratory conditions.

### Procedures

Upon arrival, hydration status was verified via urine specific gravity, (euhydration defined as urine specific gravity < 1.020) (Sawka et al. 2007; Armstrong 2007). Participants exceeding this threshold were provided with 500 mL of water and their urine specific gravity was re-evaluated after 15 min. Prior to iontophoresis, the right ventral forearm skin was inspected and, if necessary, hair was removed using a disposable razor. The skin was then cleaned using an alcohol prep pad, rinsed with deionized water, and gently dried with lint-free laboratory wipe to remove surface contaminants. Following hydration assessment, local sweat was induced on the right ventral forearm using pilocarpine iontophoresis (5 min; 0.5% pilocarpine nitrate; anode proximal, cathode 5 cm distal; Precision Hydration, Christchurch, UK). Electrodes were then removed, the skin was gently dried, and a Macroduct collector (Wescor, Logan, UT) was applied to the stimulation site. Sweat collection continued until the device was filled (~ 85 µL) or 20 min had elapsed.

After completion of iontophoresis, the left ventral forearm was prepped using the same skin cleaning procedure (alcohol prep pad, deionized water rinse, and drying with a low-lint laboratory wipe) before application of the exercise sweat collection devices (Macroduct and absorbent patch). A second Macroduct collector was affixed to the left ventral forearm for exercise sweat sampling, and an absorbent patch (Tegaderm+, 3 M, St. Paul, MN) was placed proximal to the Macroduct site for concurrent sweat collection. The contralateral forearm was used for exercise sweat collection to minimize any potential local effects of pilocarpine iontophoresis on subsequent exercise-induced sweat composition. Nude body mass was assessed using a digital scale (Health O Meter 349KLX, St. McCook, IL) and height with a wall-mounted stadiometer (Seca 216, Hamburg, Germany). Participants self-inserted a rectal thermocouple (RT-1 probe, Physitemp Instrumental Inc, Clifton, NJ) to ~ 15 cm past the anal sphincter for measurement of rectal temperature. Participants then donned a chest strap heart rate monitor (Timex, Middlebury, CT) and entered an environmental chamber (CANTROL, Canada) maintained at 27 °C and 50% relative humidity. Clothing was standardized between trials to ensure rigor.

**Fig. 1:**
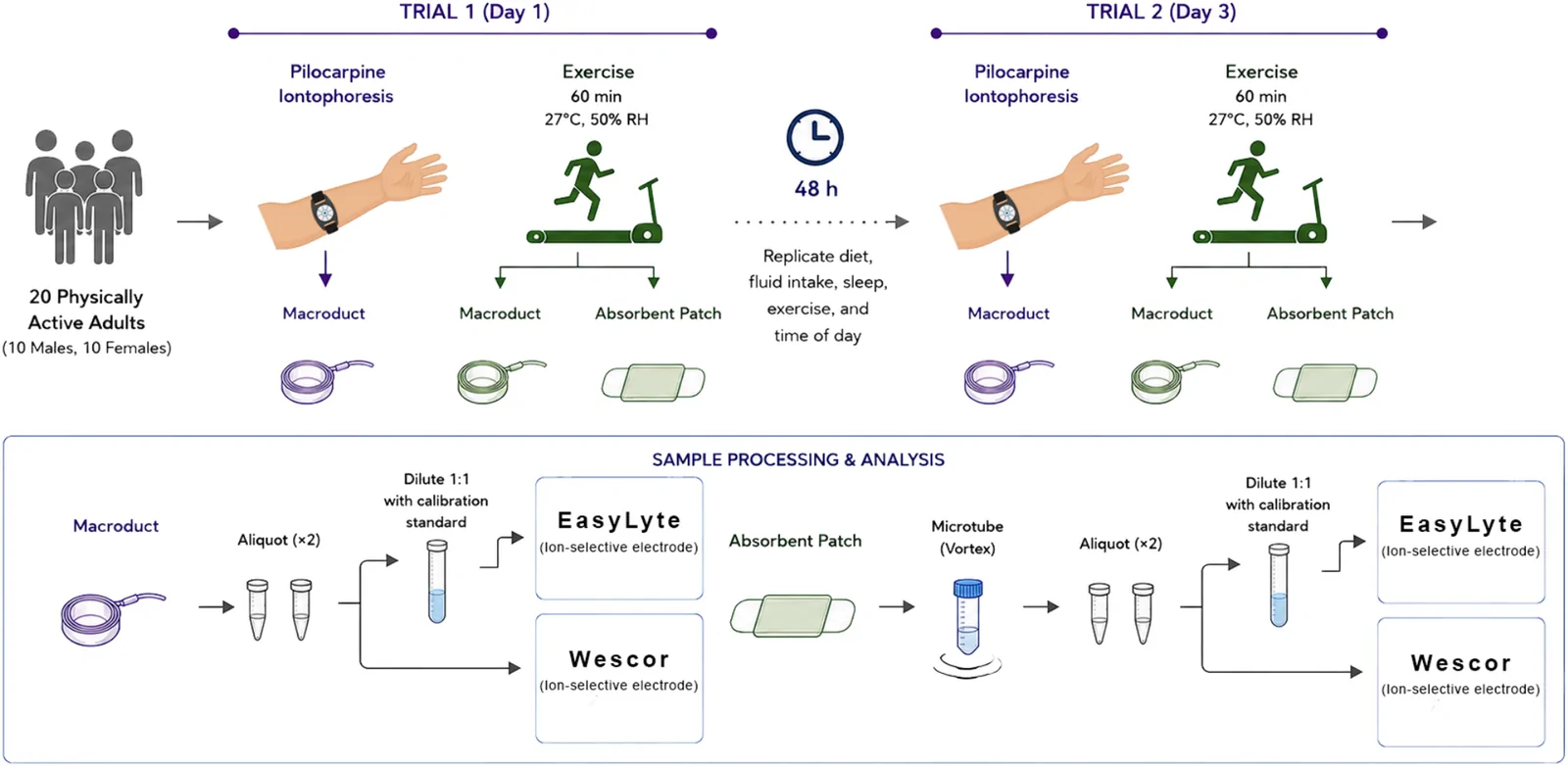
Study timeline and sample processing workflow

Participants performed 60 min of continuous exercise (either cycling *n* = 10 or treadmill running *n* = 10). Exercise intensity was self-selected at a fixed workload (speed/grade or power) that participants could maintain for 60 min. Exercise intensity was identical between trials. A self-selected workload was used to reflect the intended application of pilocarpine-based sweat testing, whereby resting sweat sodium measurements are used to guide hydration and sodium replacement strategies during an athlete’s habitual training. Measurements of rectal temperature, heart rate, rating of perceived exertion (Borg 1982), ambient temperature, and relative humidity were recorded at every 15 min. Ambient temperature and relative humidity were measured using a handheld environmental monitor (Kestrel 5400 Boothwyn, PA). No fluids were consumed during exercise. If urine was produced, it was collected and weighed. Immediately post-exercise, all instrumentation was removed, and participants towel-dried prior to a final nude body mass measurement. Whole-body sweat rate (L·h^− 1^) was calculated from the change in nude body mass before and after exercise, corrected for urine output, and normalized to exercise duration. Macroduct samples were collected until the device was filled, at which point activity was paused briefly (< 20 s) to remove the collector. Absorbent patches remained in place for the full 60-min exercise bout and were removed immediately post-exercise Fig. 1.

### Sweat sodium analysis

Sweat was collected at rest following pilocarpine iontophoresis using a Macroduct collector and during exercise using a Macroduct collector affixed to the contralateral ventral forearm, with concurrent absorbent patch-based collection (Tegaderm^+^ 3 M, St. Paul, MN) placed proximal to the Macroduct site. Macroduct samples were collected until the device was filled (~ 85µL), while patch-based sweat samples were collected over the full exercise duration and removed immediately post-exercise.

Collected sweat samples were transferred into microtubes (Eppendorf) and vortexed. A 30 µL aliquot was diluted 1:1 with a calibration standard and analyzed for sodium concentration using an ion-selective electrode analyzer (EasyLyte, Medica Corp, USA). An additional aliquot was analyzed with a Wescor 3100 Precision Hydration analyzer (Wescor, Logan, UT), which quantifies sodium concentration via ion-selective electrode technology based on the electrical potential generated by sodium activity in solution. Following each measurement, analytical lines were flushed with deionized water and air to prevent cross-contamination. Whole-body sweat sodium concentration was estimated from ventral forearm sweat sodium concentrations using regression equations previously described and cross validated by Baker et al. (Baker et al. 2009, 2020) relating regional and whole-body sweat sodium under steady-state exercise conditions.

### Data analysis

We were unable to obtain some measurements due to insufficient sweat volume or technical issues with sample collection either during pilocarpine iontophoresis or exercise. These missing data were excluded from final analyses, and the number of participants included for each variable is indicated in text, tables, and figures where appropriate. Day-to-day variability in sweat sodium concentration was evaluated by comparing Trial 1 and Trial 2 measurements obtained via pilocarpine iontophoresis (*n* = 17), exercise-derived Macroduct collection (*n* = 17), and exercise-derived absorbent patch collection (*n* = 17), separately. Agreement between measurements was assessed using Bland-Altman analysis. Typical error (TE) was calculated as the standard deviation of the paired differences divided by $\:\surd\:$2, and the coefficient of variation (CV) was calculated as pooled standard deviation divided by the overall mean and expressed as a percentage. Method agreement was examined between pilocarpine iontophoresis and exercise-derived measurements when macroduct collectors were used for both conditions (*n* = 15), as well as between analytical devices (Wescor vs. EasyLyte; *n* = 17) and between sweat collection methods (Macroduct vs. absorbent patch; EasyLyte *n* = 14, Wescor *n* = 19). Agreement between methods was evaluated using Bland-Altman analysis. Simple linear regressions were used to examine the relation between paired measurements across trials and methods. All statistical analyses were performed using R (version 2025.05.1 + 513; R Foundation for Statistical Computing, Vienna, Austria) and GraphPad Prism (version 10.6.1; GraphPad Software, San Diego, CA). Descriptive data are presented as mean ± standard deviation (SD).

## Results

Pre-trial hydration status and dietary intake are presented in Table 1, while physiological responses during exercise are shown in Table 2.

**Table 2:** Physiological responses during Trial 1 and Trial 2. Values are presented as mean ± SD

	Trial 1	Trial 2	*p* Value
Pre rectal temperature (°C)	37.25 ± 0.20	37.29 ± 0.43	0.734
Post rectal temperature (°C)	38.59 ± 1.25	38.58 ± 0.55	0.276
Average rectal temperature (°C)	38.00 ± 0.46	38.09 ± 0.42	0.317
Average heart rate (bpm)	141 ± 13	141 ± 13	0.972
Average RPE (Borg 6–20)	11 ± 1	11 ± 2	0.929
Sweat rate (mL/h)	1065 ± 394	1085 ± 418	0.755
Sweat rate (mL/kg/h)	14.9 ± 4.7	15.2 ± 4.6	0.797

### Day-to-day variability of sweat sodium concentration

**Fig. 2:**
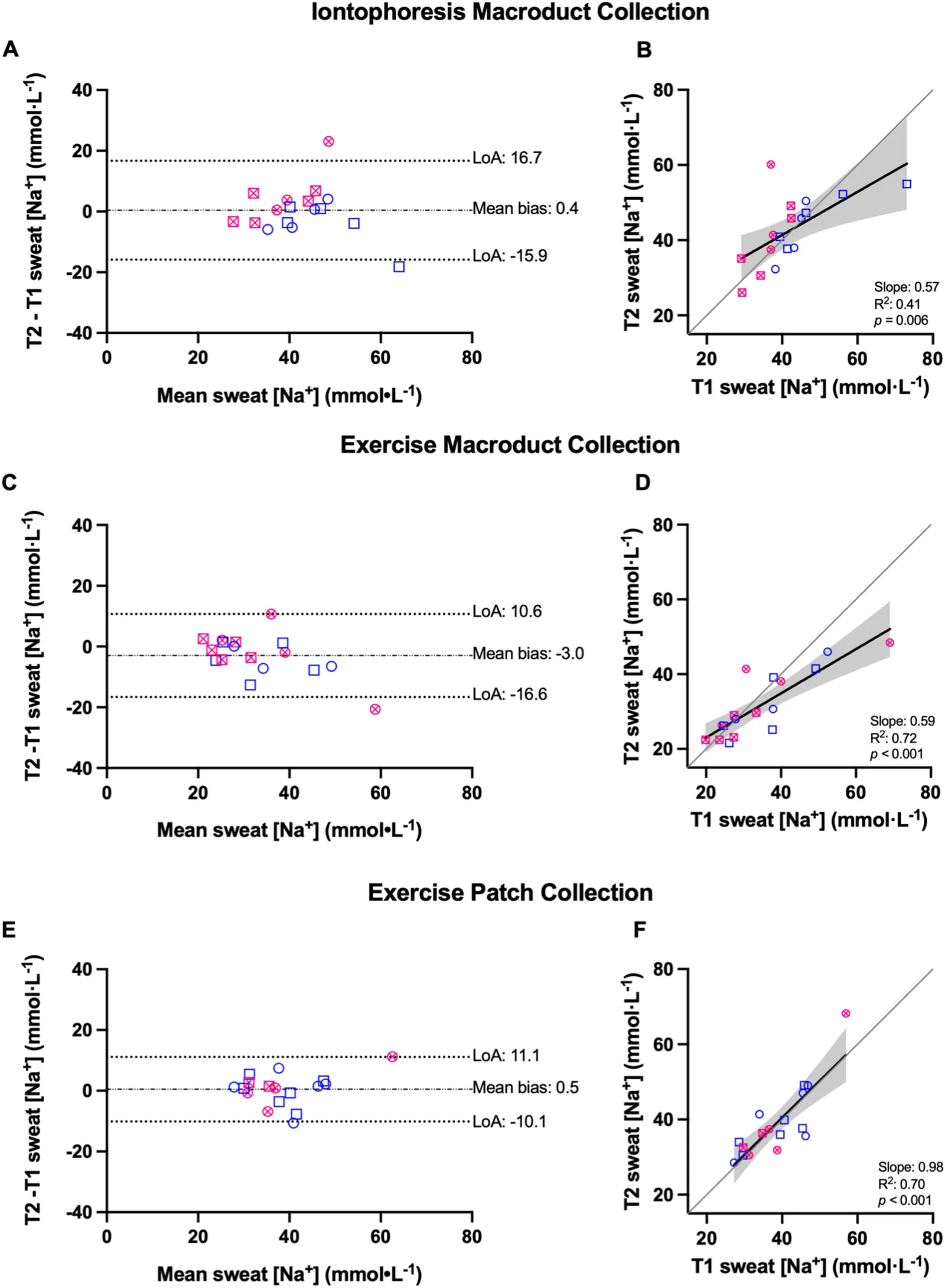
Day-to-day variability of sweat sodium concentration assessed by pilocarpine iontophoresis and exercise-induced sweating. Bland-Altman plots and linear regression analysis show agreement between Trial 1 (T1) and Trial 2 (T2) measurements for pilocarpine iontophoresis with Macroduct collection (A and B; n=17), exercise-derived sweat collected with Macroduct (C and D; n=17, and exercise-derived sweat collected with absorbent patches (E and F; n=17). Circles denote treadmill running and squares show cycling. Females are shown in hatched pink symbols and males are shown in open blue symbols.

Figure 2 illustrates day-to-day variability in sweat sodium concentrations assessed via pilocarpine iontophoresis and exercise-induced sweating. Mean sweat sodium concentration for iontophoresis with Macroduct collection was 42.3 ± 10.3 mmol·L^− 1^ in trial 1 and 42.7 ± 9.2 mmol·L^− 1^ in Trial 2 (*n* = 17). The mean bias between trials (trial 2-trial 1) was 0.4 mmol·L^− 1^ [95% limits of agreement:−15.9 to 16.7 mmol·L^− 1^]. The typical error was 5.9 mmol·L^− 1^ and the coefficient of variation was 13.8%. Linear regression yielded a slope of 0.57 (R^2^ = 0.41, *p* =.006), with a standard error of the estimate of 7.3 mmol·L^− 1^.

For exercise-derived sweat collected via Macroduct, mean sweat sodium concentration was 34.7 ± 12.7 mmol·L^− 1^ in Trial 1 and 31.7 ± 8.8 mmol·L^− 1^ in Trial 2 (*n* = 17). The mean bias between trials was − 3.0 mmol·L^− 1^ [−16.6 to 10.6 mmol·L^− 1^]. The typical error was 4.9 mmol·L^− 1^ and the coefficient of variation was 14.9%. Linear regression yielded a slope of 0.59 (R^2^ = 0.72, *p* <.001), with a standard error of the estimate of 4.8 mmol·L^− 1^. Mean sweat sodium concentration for exercise-derived sweat collected using absorbent patches, was 38.7 ± 8.3 mmol·L^− 1^ in Trial 1 and 39.1 ± 9.8 mmol·L^− 1^ in Trial 2 (*n* = 17). The mean bias between trials was 0.5 mmol·L^− 1^ [−10.1 to 11.1]. The typical error was 3.8 mmol·L^− 1^ and the coefficient of variation was 9.9%. Linear regression yielded a slope of 0.98 (R^2^ = 0.69, *p* <.001), with a standard error of the estimate of 5.6 mmol·L^− 1^.

### Iontophoresis and exercise sweat sodium concentration

**Fig. 3:**
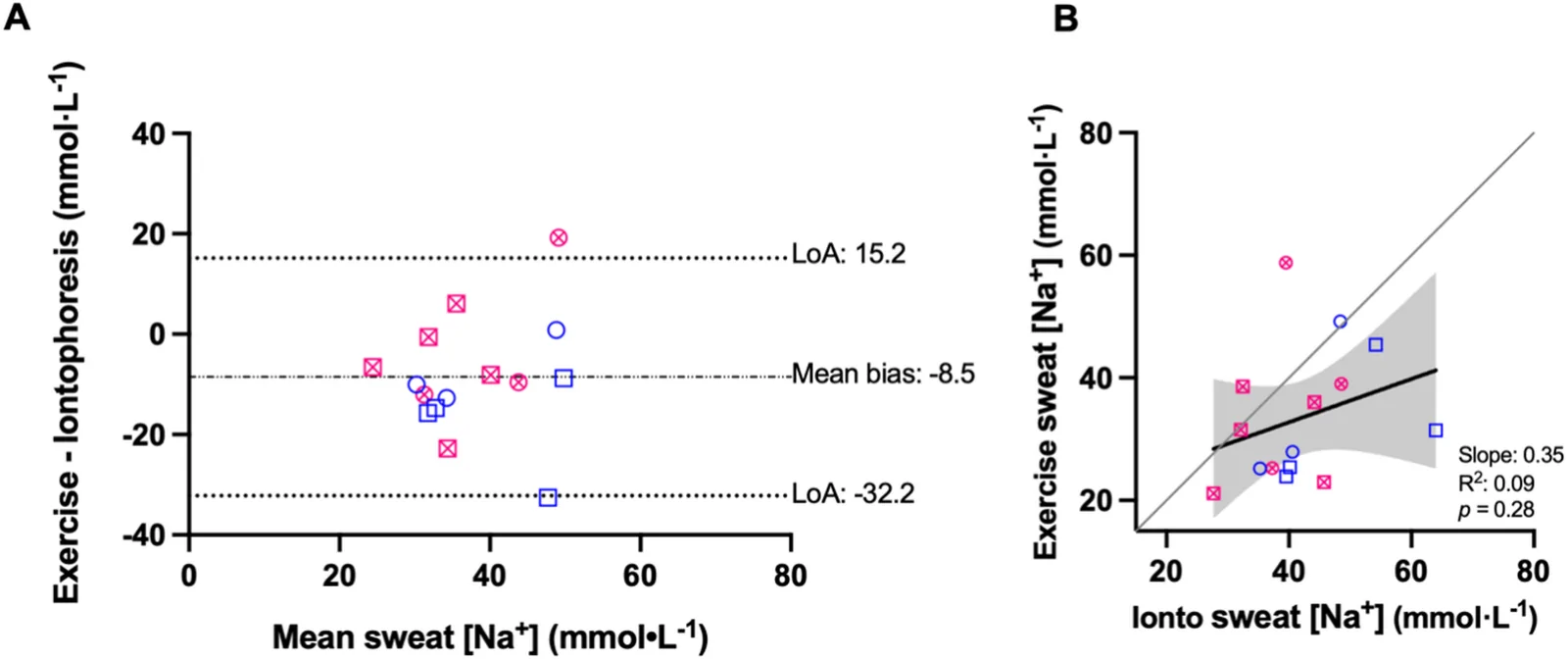
Agreement between pilocarpine iontophoresis-derived and exercise-derived sweat sodium concentration. Panel A presents the Bland-Altman plot and panel B shows the linear regression analysis. Sweat samples were collected using Macroduct collectors and analyzed using the EasyLyte analyzer. N=15. Circles denote treadmill running and squares show cycling. Females are shown in hatched pink symbols and males are shown in open blue symbols.

The agreement between sweat sodium concentrations obtained via pilocarpine iontophoresis and those measured during exercise is shown in Fig. 3. When Macroduct was used for both iontophoresis and exercise sweat collection, mean sweat sodium concentration was 42.0 ± 9.31 mmol·L^−1^ for iontophoresis and 33.5 ± 11.0 mmol·L^−1^ for exercise (*n* = 15). The mean bias (exercise – iontophoresis) was − 8.5 mmol·L^−1^ [−32.2 to 15.2 mmol·L^−1^]. Linear regression yielded a slope of 0.35 (R^2^ = 0.09, *p* =.28), with a standard error of the estimate of 10.9 mmol·L^−1^

### Macroduct and patch collection methods

**Fig. 4:**
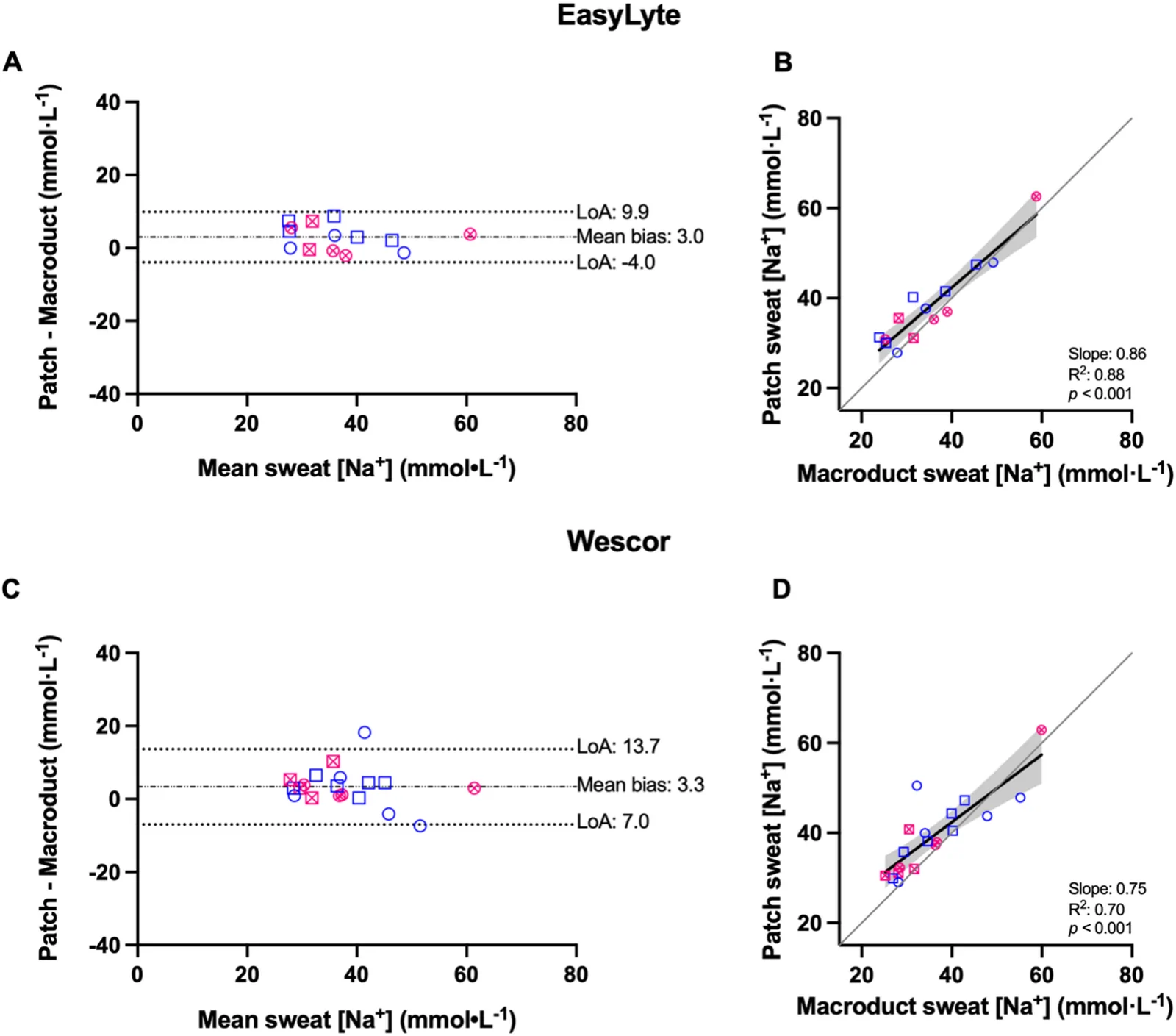
Agreement between Macroduct and absorbent patch sweat collection methods during exercise. Bland-Altman plots and linear regression analyses show the agreement between exercise-derived sweat sodium concentrations collected using Macroduct and absorbent patch methods when samplings were analyzed using the EasyLyte (**A **and **B**; n=14) and Wescor (**C **and **D**; n=19) analyzers. Circles denote treadmill running and squares show cycling. Females are shown in hatched pink symbols and males are shown in open blue symbols.

Figure 4 illustrates the agreement between exercise-derived sweat sodium concentrations collected using Macroduct and absorbent patch methods. When analyzed using the EasyLyte, mean exercise sweat sodium concentrations were 35.4 ± 10.1 mmol·L^− 1^ for Macroduct collection and 38.3 ± 9.3 mmol·L^− 1^ for patch collection (*n* = 14). The mean bias (patch – Macroduct) was 3.0 mmol·L^− 1^ [−4.0 to 9.9 mmol·L^− 1^]. Linear regression yielded a slope of 0.86 (R^2^ = 0.88, *p* <.001), with a standard error of the estimate of 3.4 mmol·L^− 1^. When analyzed using the Wescor, mean exercise sweat sodium concentration was 36.2 ± 9.6 mmol·L^− 1^ for Macroduct collection and 39.6 ± 8.6 mmol·L^− 1^ for patch collection (*n* = 19). The mean bias was 3.3 mmol·L^− 1^[−8.0 to 13.7 mmol·L^− 1^]. Linear regression yielded a slope of 0.75 (R^2^ = 0.70, *p* <.001), with a standard error of the estimate of 4.8 mmol·L^− 1^

### Wescor and easylyte analyzers

**Fig. 5:**
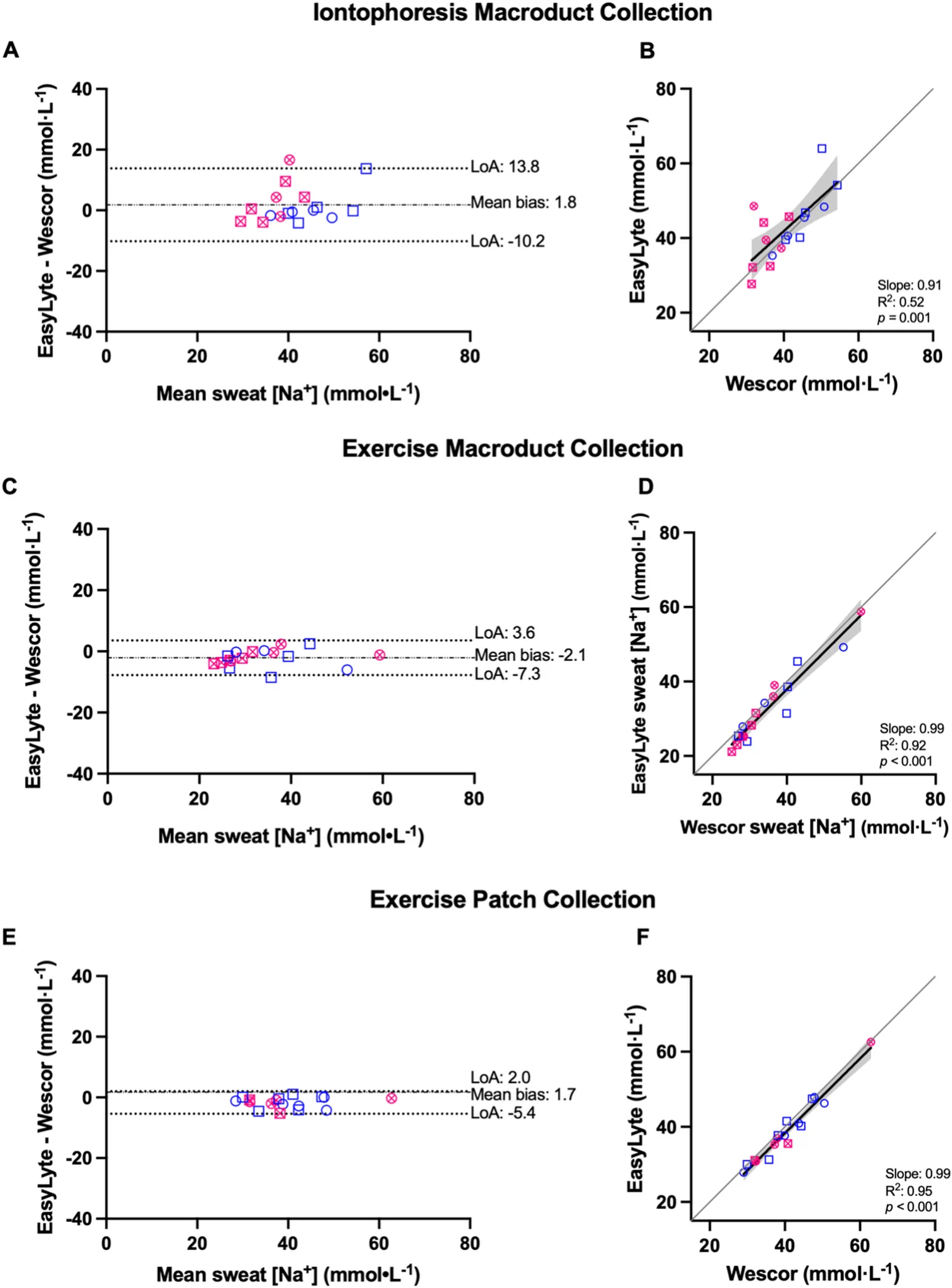
Agreement between Wescor and EasyLyte sweat sodium analyzers across sampling methods. Bland-Altman plots and linear regression analyses show the agreement between sodium concentrations measured using the Wescor and EasyLyte analyzers for iontophoresis-derived sweat collected using Macroduct (**A **and **B**), exercise-derived sweat collected using Macroduct (**C **and**D**), and exercise-derived sweat collected using absorbent patch (E and F). Circles denote treadmill running and squares show cycling. Females are shown in hatched pink symbols and males are shown in open blue symbols.

Figure 5 illustrates the agreement between sweat sodium concentrations measured using the Wescor and EasyLyte analyzers. For iontophoresis-derived sweat collected via macroduct, mean sweat sodium concentration was 40.7 ± 7.0 mmol·L^− 1^ when analyzed using the Wescor and 42.5 ± 8.8 mmol·L^− 1^ when analyzed using the EasyLyte (*n* = 17). The mean bias (EasyLyte – Wescor) was 1.8 mmol·L^− 1^ [−10.2 to 13.8]. Linear regression yielded a slope of 0.91 (R^2^ = 0.52, *p* =.001), with a standard error of the estimate of 6.3 mmol·L^− 1^. For exercise-derived sweat collected using absorbent patches, mean sweat sodium concentration was 40.6 ± 8.5 mmol·L^− 1^ using the Wescor and 38.9 ± 8.7 mmol·L^− 1^ using the EasyLyte (*n* = 17). The mean bias was − 1.7 mmol·L^− 1^ [−5.4 to 2.0 mmol·L^− 1^]. Linear regression analysis yielded a slope of 0.99 (R^2^ = 0.95, *p* <.001), with a standard error of the estimate of 2.0 mmol·L^− 1^. For exercise-derived sweat collected via Macroduct, mean sweat sodium concentration was 35.3 ± 9.9 mmol·L^− 1^ using the Wescor and 33.2 ± 10.4 mmol·L^− 1^ using the EasyLyte (*n* = 17). The mean bias was − 2.1 mmol·L^− 1^ [−7.8 to 3.6 mmol·L^− 1^]. Linear regression yielded a slope of 0.99 (R^2^ = 0.92, *p* <.001), with a standard error of the estimate of 3.0 mmol·L^− 1^

## Discussion

The purpose of this study was to evaluate the reliability of sweat sodium assessment and determine the influence of common sweat induction, sweat collection, and analytical approaches on sweat sodium concentration measurements in physically active adults. The primary findings were as follows: (1) Moderate day-to-day variability was observed across all conditions, with coefficients of variation ranging from ~ 10–15%. Importantly, this variability was similar for iontophoresis and exercise-derived measures. (2) Sweat sodium concentration derived from iontophoresis was modestly higher than exercise (mean bias 8.5 mmol·L^− 1^). (3) Collection method (Macroduct vs. absorbent patch) showed good agreement overall, with only modest differences in exercise-derived concentrations; and (4) Analytical device differences were negligible, indicating very strong agreement between measurement systems. Collectively, these findings improve our understanding of the expected biological and methodological variability in sweat sodium assessment and provide important context for interpreting sweat sodium measurements used to develop individualized hydration and sodium replacement strategies.

A number of biological and behavioral factors, including heat acclimation, hydration status, and dietary sodium intake, may contribute to intra-individual fluctuations in sweat sodium concentrations in both laboratory and field settings (Kirby and Convertino 1986; McCubbin et al. 2019; Baker et al. 2022). Likewise, methodological factors, including sweat collection technique and analytical approach are known to contribute to measurement variability (Baker et al. 2009; Dziedzic et al. 2014). Thus, it is unsurprising that even under standardized conditions, repeated assessments rarely yield identical values. In the present study, we observed modest trial-to-trial variability in both iontophoresis- and exercise-derived sweat sodium concentrations. Typical error ranged from approximately 4–6 mmol·L^− 1^, with coefficients of variation near 10–15% depending on the methodological approach. The limits of agreement indicated that repeated measures within an individual could differ by approximately ± 10–17 mmol·L^− 1^. Importantly, these differences persisted despite matched diet, hydration status, exercise intensity, and environmental conditions. Thus, this variability reflects the combined influence of normal physiological fluctuation and inherent measurement error (i.e., noise) rather than poor methodological design. Notably, the magnitude observed here is consistent with prior reports of intra-individual variability in sweat sodium concentrations (Baker et al. 2022). Importantly, the present findings extend this literature by demonstrating comparable variability between pilocarpine-induced and exercise-induced sweating under matched laboratory conditions.

Pharmacological sweat induction via pilocarpine iontophoresis has gained popularity in applied sport settings due to its speed and practicality; however, its translation to exercise conditions remains unclear. Pilocarpine stimulates eccrine glands locally through cholinergic activation (Gibson and Cooke 1959), whereas exercise-induced sweating reflects integrated central and peripheral thermoregulatory control influenced by metabolic heat production, skin blood flow, and systemic physiological strain (Nadel et al. 1971). Recently Harris et al.(2026) reported intensity-dependent agreement between iontophoresis- and exercise-derived sweat sodium concentration under thermoneutral conditions, suggesting that agreement may vary according to exercise intensity. In the present study, exercise-derived sweat sodium concentrations were lower than iontophoresis-derived values under the conditions tested, with wide limits of agreement between methods (95% LoA: −32.2 to 15.5 mmol·L^− 1^). Although mean bias was modest (−8.5 mmol·L^− 1^, ~ 195 mg·L), the wide limits of agreement indicate that the two approaches are not interchangeable at the individual level. Several factors may contribute to this disagreement. For example, exercise-induced increases in sweat rate and recruitment of active sweat glands influence ductal sodium reabsorption and final sweat composition (Sato 1977), whereas pilocarpine stimulates a localized region under resting conditions (Gibson and Cooke 1959), potentially altering sweat rate and ductal transit time compared with whole-body exercise. Thus, differences in regional activation, sweat flow dynamics, and thermoregulatory drive may contribute to systematic variations between methods. The present findings are generally consistent with previous observations of intensity-dependent agreement between pilocarpine- and exercise-derived sweat sodium concentrations (Harris et al. 2026). However, the present study extends this work by evaluating agreement during longer-duration exercise in a warm-humid environment while simultaneously accounting for the influence of sweat collection methodology and analytical device selection. Collectively, these data suggest that both physiological and methodological factors should be considered when interpreting sweat sodium measurements obtained using pilocarpine iontophoresis.

Sweat collection methodology can influence measured sodium concentration due to differences in sampling duration, regional pooling, evaporation, and potential contamination (Baker et al. 2009; Dziedzic et al. 2014). In the present study, exercise-derived Macroduct samples were collected until the device was filled, whereas absorbent patches remained in place throughout the 60-minute exercise bout. Consequently, the two methods differed in both collection duration and sampling conditions, which may have contributed to the observed differences in sweat sodium concentration. Absorbent patch techniques typically collect sweat over longer durations and larger surface areas, whereas Macroduct systems sample smaller, localized volumes over shorter time intervals. Although differences in sampling duration and the local microclimate may influence sweat sodium concentration, the Tegaderm dressing is waterproof and largely vapor resistant, minimizing the likelihood of evaporative concentration. In the present study, exercise-derived sweat sodium concentrations obtained via absorbent patch were modestly higher than those collected via Macroduct. Consistent with this, linear regression analysis demonstrated a strong relationship between absorbent patch and Macroduct measurements (slope = 0.86, R^2^ = 0.88), indicating strong agreement between collection methods. From a practical perspective, Macroduct collection requires maintaining an intact seal and uninterrupted capillary flow; device displacement, loss of seal, or premature filling may compromise sample integrity. These practical challenges may be amplified during dynamic exercise such as running.

Ion-selective electrode analyzers are widely used for sweat sodium assessment; however, small inter-device variation has been reported depending on calibration procedures, sample handling, and dilution protocols (Barnes et al. 2019; Baker 2017). Consistency in analytical technique is therefore recommended when conducting repeated measurements. Our data show good agreement and relatively small differences between the Wescor and EasyLyte analyzers using both exercise- and iontophoresis-derived sweat sodium concentration. While the limits of agreement were slightly wider for iontophoresis-derived samples, the magnitude of inter-device bias was modest relative to the biological variability observed between trials. These findings suggest that both analytical systems provide comparable sweat sodium estimates under controlled laboratory conditions. However, consistency in analyzer selection remains important for longitudinal monitoring, as switching devices may introduce avoidable methodological variability into sodium-loss calculations.

Based on these findings we suggest that athletes, coaches, and practitioners interpret a single sweat sodium value as an estimate within an expected physiological range rather than a fixed constant. While the difference in sweat sodium concentration found herein were modest, they are not without consequence. For example, at the mean sweat rates observed in this study (~ 1.1 Lh^− 1^), a 5 mmol·L^− 1^ difference corresponds to approximately 110–120 mg of sodium per hour, which may accumulate to a meaningful deficit or surplus during prolonged or ultra-endurance events. Accordingly, we suggest that individual sodium replacement strategies should account for expected biological variability rather than rely on a single-point estimate. In addition, iontophoresis-derived values may not directly reflect sodium losses during prolonged exercise, and practitioners should therefore view iontophoresis results as starting points for refining sodium replacement strategies during training prior to competition. Finally, consistency in sweat collection method is important when monitoring athletes longitudinally, as switching techniques may introduce additional variability beyond normal biological fluctuation.

A few limitations should be acknowledged. First, sweat sodium concentration was measured regionally at the ventral forearm and used to estimate whole-body sodium concentration. Although this approach is supported by prior validation equations (Baker et al. 2020), regional sampling inherently introduces prediction error and may not fully capture whole-body variability during prolonged exercise. To address this limitation, all primary analyses were repeated using raw regional forearm sweat sodium concentrations (Supplementary Tables S1-S4). Although absolute values and limits of agreement differed modestly, the overall interpretation of the findings was unchanged. Second, exercise intensity was self-selected and fixed within participants rather than standardized to metabolic heat production or percentage of maximal aerobic capacity. Although this approach reflects applied training and racing conditions, sweat rate and glandular sodium reabsorption are influenced by exercise intensity and metabolic heat production (Nadel et al. 1971; Sato 1977). Variability in relative physiological strain between participants may therefore have contributed to differences in sweat composition and method agreement. Third, menstrual cycle phase was not recorded or controlled in the present study. Although participants completed both trials within 48 h to minimize within-subject physiological variation, hormonal fluctuations associated with the menstrual cycle may have contributed to some of the observed variability. Finally, although participants were instructed to replicate diet and fluid intake between trials and compliance was monitored, dietary sodium intake was not prescribed or provided. Sodium status influences sweat sodium concentration (Baker et al. 2022; McCubbin et al. 2019), and tighter dietary control may have further reduced intra-individual variability. Future research should examine method agreement across broader intensities, environmental conditions, and exercise durations, particularly during prolonged efforts that include fluctuations in workload and environmental stress similar to real-world endurance and ultra-endurance competition. Such work may clarify how dynamic changes in sweat rate and thermoregulatory strain influence method agreement over time.

## Conclusion

Sweat sodium concentration demonstrates meaningful day-to-day variability under controlled laboratory conditions, with comparable fluctuation observed between pilocarpine-induced and exercise-induced assessments. Exercise-derived values were lower than iontophoresis-derived values, and both collection method and analytical device contributed modest methodological differences. Although these effects were small in isolation, they may meaningfully influence sodium-loss estimates when extrapolated across prolonged endurance exercise. Sweat testing remains a valuable tool for individualized hydration planning; however, results should be interpreted within an expected range of biological and methodological variability, and consistency in testing approach is essential for practical application.

## Supplementary Information

Below is the link to the electronic supplementary material.

Supplementary Material 1

## Data Availability

The data that support the findings of this study are available from the authors on reasonable request.
