# Supplementary material for: Day-to-day variability and method agreement of sweat sodium concentration during pilocarpine iontophoresis and exercise
Source: Eur J Appl Physiol. 2026 Aug 27;126(11):6341–52. doi: 10.1007/s00421-026-06409-0 (PMC13751564; doi:10.1007/s00421-026-06409-0)
Supplement: Supplementary file 1 [file 421_2026_6409_MOESM1_ESM.docx]

**Table S1.** Day-to-Day Reliability of Raw Regional Forearm Sweat Sodium Concentration

| **Comparison** | **n** | **Mean T1 ± SD (mmol·L^-1^)** | **Mean T2 ± SD (mmol·L^-1^)** | **Mean Bias (T2-T1)** | **Typical Error (mmol·L^-1^)** | **CV (%)** | **Lower 95% LoA** | **Upper 95% LoA** |
| --- | --- | --- | --- | --- | --- | --- | --- | --- |
| **Iontophoresis** | 17 | 47.49 ± 17.43 | 48.19 ± 15.61 | 0.71 | 9.97 | 20.9 | -26.94 | 28.35 |
| **Exercise Macroduct** | 17 | 34.61 ± 21.50 | 29.57 ± 15.00 | -5.04 | 8.37 | 26.1 | -28.25 | 18.17 |
| **Exercise Patch** | 17 | 42.18 ± 16.62 | 41.35 ± 14.06 | -0.82 | 6.51 | 15.6 | -18.86 | 17.21 |

Values were repeated using raw regional forearm sweat sodium concentrations. Analyses were reported to evaluate whether conversion to

estimated whole-body sweat sodium concentration influenced interpretation of the findings reported in the primary manuscript. Interpretation

was unchanged across all conditions.

**Table S2.** Agreement Between Sweat Induction Approaches Using Raw Regional Forearm Sweat Sodium Concentrations

| **Comparison** | **n** | **Mean Iontophoresis ± SD (mmol·L^-1^)** | **Mean Exercise ± SD (mmol·L^-1^)** | **Mean Bias (Exercise - Iontophoresis) (mmol·L^-1^)** | **Lower 95% LoA** | **Upper 95% LoA** |
| --- | --- | --- | --- | --- | --- | --- |
| **Iontophoresis Macroduct vs Exercise Macroduct** | 15 | 47.01 ± 15.80 | 30.08 ± 14.19 | -16.93 | -51.83 | 17.97 |
| **Iontophoresis Macroduct vs Exercise Patch** | 14 | 42.48 ± 8.82 | 33.20 ± 10.35 | -9.95 | -36.18 | 16.27 |

Exercise-derived sweat sodium concentrations remained lower than iontophoresis-derived values, and interpretation was unchanged.

**Table S3.** Agreement Between Sweat Collection Methods Using Raw Regional Forearm Sweat Sodium Concentrations

| **Comparison** | **n** | **Mean Macroduct ± SD (mmol·L^-1^)** | **Mean Patch ± SD (mmol·L^-1^)** | **Mean Bias (Patch - Macroduct) (mmol·L^-1^)** | **Lower 95% LoA** | **Upper 95% LoA** |
| --- | --- | --- | --- | --- | --- | --- |
| **Wescor Analyzer** | 19 | 37.24 ± 16.29 | 42.89 ± 14.59 | 5.66 | -11.88 | 23.19 |
| **Medica (EasyLyte) Analyzer** | 14 | 35.76 ± 17.20 | 40.76 ± 15.85 | 5.01 | -6.72 | 16.73 |

Absorbent patch values remained modestly higher than Macroduct values and interpretation was unchanged.

**Table S4.** Agreement Between Analytical Devices Using Raw Regional Forearm Sweat Sodium Concentrations

| **Comparison** | **n** | **Mean Wescor ± SD (mmol·L^-1^)** | **Mean Medica ± SD (mmol·L^-1^)** | **Mean Bias (Medica - Wescor) (mmol·L^-1^)** | **Lower 95% LoA** | **Upper 95% LoA** |
| --- | --- | --- | --- | --- | --- | --- |
| **Iontophoresis Samples** | 17 | 44.79± 11.92 | 47.84 ± 14.97 | 3.05 | -17.35 | 23.44 |
| **Exercise Macroduct Samples** | 17 | 35.68 ± 16.87 | 32.09 ± 17.57 | -3.59 | -13.21 | 6.04 |
| **Exercise Patch Samples** | 17 | 44.65 ± 14.44 | 41.76 ± 14.69 | -2.88 | -9.18 | 3.42 |

Agreement between analytical devices remained good across all sampling approaches and interpretation was unchanged.
